# Orthodontic Space Management in Pediatric Dentistry: A Clinical Review

**DOI:** 10.7759/cureus.76026

**Published:** 2024-12-19

**Authors:** Abdullah Koaban, Shahad S Alotaibi, Khalid M Abu Nakha, Sakhaa Bin Huraib, Miqdad H Alhassan, Abdullah R Rubayan, Kawther Z Alzahir, Latifa A Alhamdan, Ziad A Alshehri, Solara M Hamza, Bushra A Alqahtani

**Affiliations:** 1 Orthodontics and Dentofacial Orthopedics, Ministry of Health, First Health Cluster, Riyadh, SAU; 2 Dentistry, Princess Nourah bint Abdulrahman University, Riyadh, SAU; 3 Dentistry, Ministry of Health, Riyadh, SAU; 4 Dentistry, Nova Dental Clinics, Riyadh, SAU; 5 Dentistry, King Faisal General Hospital, Hofuf, SAU; 6 Dentistry, University of Hail, Hail, SAU; 7 Dentistry, Imam Abdulrahman Bin Faisal University, Dammam, SAU; 8 Dentistry, King Faisal Hospital, Makkah, SAU; 9 Dentistry, King Khalid University, Abha, SAU; 10 Dentistry, Khamis Mushait Specialized Dental Center, Khamis Mushait, SAU; 11 Dentistry, Armed Forces Hospital Southern Region, Abha, SAU

**Keywords:** mixed dentition, primary dentition, space maintainer, space management, space regainer

## Abstract

The mixed dentition stage is a vital period characterized by significant physiological changes, including jaw growth, the development and eruption of permanent teeth, the exfoliation of primary teeth, and the maturation of surrounding soft tissues. These processes collectively ensure functional, esthetic, and stable occlusion. Disruptions during this stage, such as the premature loss of deciduous teeth, can lead to spacing or crowding issues and affect the dental arch length and the position of permanent teeth. To prevent or mitigate malocclusion, space maintainers and regainers are commonly used as part of space management strategies during the mixed dentition phase. This clinical review examines traditional and modern approaches to space management, highlighting the types of appliances used and their effectiveness. It emphasizes the importance of preserving primary teeth until their natural exfoliation, as they serve as the best natural space maintainers. In cases of premature tooth loss, removable or fixed space maintainers and regainers are effective tools to minimize malocclusion and ensure optimal dental outcomes.

## Introduction and background

The mixed dentition stage is a vital period involving significant physiological processes, including jaw growth, the development of permanent tooth buds, the physiological resorption of primary teeth, the remodeling of surrounding alveolar bone, and the maturation of soft tissues. During this stage, permanent teeth must replace primary teeth promptly to ensure proper jaw development with the establishment of good occlusion [1].

However, managing space-related issues poses a significant challenge for pediatric dentists, particularly in cases of early loss of primary teeth [1,2]. Primary teeth are integral to eating, speaking, and esthetics. They also serve as natural space maintainers, guiding the eruption of permanent teeth [3].

The early loss of primary teeth can lead to adjacent teeth migrating into the extraction space, reducing the arch perimeter and compromising the eruption of permanent teeth [4]. Patano et al. demonstrated that unilateral loss of a primary canine or first molar can result in a significant midline shift and mesial drifting of the buccal segment, highlighting the importance of space conservation [5]. To prevent or minimize malocclusion, space maintainers or regainers are commonly used as initial treatments during the mixed dentition stage [2]. Guo et al. emphasized that effective space management during this transitional phase is critical to maintaining proper occlusion and preventing long-term complications [1].

Space management is a fundamental aspect of pediatric dentistry, and it involves addressing the challenges of early tooth loss, crowding, and arch discrepancies. Conventional space maintainers, such as band and loop and Nance appliances, have been proven effective in preserving arch length and guiding the eruption of permanent teeth. However, advancements in space regainers, such as lip bumpers, pendulum appliances, and fiber-reinforced composites, have broadened the scope of interceptive orthodontics by allowing space to be created in cases where it has already been lost. Emerging technologies, including 3D printing and computer-aided design/manufacturing (CAD/CAM), have introduced patient-specific, precision-engineered appliances that reduce chair-side time and improve treatment outcomes. Furthermore, the integration of artificial intelligence (AI) in treatment planning and patient education represents a promising step toward "smart" pediatric dentistry. This review explores a wide array of traditional and innovative approaches to space maintainers and regainers, evaluating their clinical efficacy and limitations. It also examines the evolving role of modern technologies in advancing space management strategies.

## Review

Methodology

This review provides an overview of the extensive research based on the management of space-related problems in pediatric patients. A manual literature search was carried out using databases such as PubMed, Google Scholar, ScienceDirect, Scopus, and Cereus with specific keywords, “space management,” “space maintainer,” “space regainer,” “primary dentition,” “mixed dentition,” and “permanent dentition.” The extant literature spanning from the years 2000 to 2024 was evaluated and comprised systemic reviews, case studies, longitudinal studies, and questionnaire-based surveys. Only recent articles relevant to pediatric dentistry and space problems and those within the scope of this review were included. Only studies published in the English language were included. Studies in languages other than English, studies with only abstracts available, and studies beyond the scope of this review were excluded.

Discussion

Aetiology of Space Problems

The untimely loss of primary molars is the most common etiology related to space problems in pediatric patients, as it causes permanent teeth to drift [6,7]. A cross-sectional study conducted on school-going children aged five to nine years in Melmaruvathur, Tamil Nadu, India, found that 20.8% of the children had lost their primary teeth before the age of six years, with males being more commonly affected than females. Among the different types of tooth loss, primary molars (98.2%) were the most frequently lost teeth, followed by incisors (1.5%) and cuspids (0.3%). The left lower primary first molars (42.3%) were the most often missing teeth, and the frequency of loss was highest in eight-year-old children (38.9%). The early loss of primary molars was highly prevalent and led to arch discrepancies and progression toward malocclusion. This finding emphasizes the need for pediatric dentists to detect such issues early and initiate appropriate space management measures [6].

Lin et al. conducted a study involving 19 children with unilateral loss of a maxillary primary molar, using the intact contralateral side as a control [7]. The study found that the initial D+E space at the extraction site (16.70 ± 0.69 mm) was significantly reduced six months after tooth removal (15.62 ± 1.13 mm), compared to the space on the control side (16.88 ± 1.12 mm). Additionally, a shorter arch length (25.47 ± 1.58 mm) and a larger inter-canine width (31.29 ± 2.49 mm) were recorded six months post-extraction compared to the initial arch length (25.66 ± 1.64 mm) and inter-canine width (30.42 ± 2.64 mm). These findings demonstrate that early loss of primary molar results in maxillary arch changes, including distal tipping of primary canines and palatal migration of maxillary incisors, leading to a 1 mm loss of space [7].

Kaklamanos et al. combined data from different controlled studies to investigate the impact of early loss of primary molars on dental arch spatial changes [8]. Only two analyzable split-mouth studies were included, focusing on the premature loss of mandibular molars. The findings revealed greater space loss at two, four, six, and eight-month follow-ups, with a -1.5 mm difference observed during the final examination (95% confidence interval: -2.080 to -0.925; p = 0.000; random effects model). The authors emphasized the importance of individualized management and the need to make a diagnosis before proceeding with space management [8]. Additionally, a lack of space in the arch and a decrease in the arch perimeter, particularly in the mandibular region, can lead to crowding [9]. Crowding has a strong hereditary tendency, and it can be classified as primary, secondary, or tertiary. Primary crowding has a genetic predilection and involves tooth material and arch length discrepancies. Secondary crowding occurs due to the premature loss of primary teeth, especially molars, resulting in shorter arch lengths. Tertiary crowding, also known as late lower incisor crowding, occurs toward the end of the mandibular growth peak [10]. Crowding should be managed promptly, as it can worsen over time [5,11].

Evaluation and Diagnosis of Arch Space Discrepancies

The premature loss of teeth can lead to significant disturbances in arch integrity and misalignment of permanent teeth. While space maintainers or regainers can be effective, their use must be evaluated on a case-by-case basis through thorough patient examination.

A longitudinal study was undertaken to investigate dental arch space changes following the unilateral premature loss of a primary molar. The study spanned 12 months and involved 13 children with a mean age of 6.0 ± 0.74 years at the time of extraction. Maxillary study dental casts were obtained from patients two to three days after tooth loss and again at a follow-up appointment 12 months later. Six reference lines were marked and measured on the dental casts as follows: D+E space, arch width, arch length, inter-canine width, inter-canine length, and arch perimeter. For each participant, the D+E space of the contralateral intact side served as a control. A t-test was used to compare D+E space changes with those of the control group, while a paired t-test was employed to compare case measurements between the initial examination and the 12-month follow-up. The study concluded that space changes after 12 months were primarily due to the distal drift of the primary canine toward the extraction site. Mesial tipping of the permanent molar did not occur, but the arch length and inter-canine width increased. The selected cases of premature loss of deciduous molars did not require the use of space maintainers [12].

Thus, a combined intra-oral and extra-oral examination is necessary to determine the diagnosis and quantify space loss or requirements for normal dentition function. As a general rule, a lack of space in primary dentition is considered a predictor of crowding in permanent dentition, necessitating early space management. This situation can also be associated with "closed" primary dentition, which prevents the mesial shift of erupting permanent molars into a class I molar relationship [13].

To diagnose the lack of space in primary dentition as an indicator of crowding, dentists typically obtain measurements using plaster models with a digital caliper before and after treatment. The measure of crowding depends on the discrepancy between the tooth size and the arch length. Tooth size is calculated as the sum of the mesiodistal diameters of all teeth, while arch length is defined as the perpendicular distance between a line connecting the mesial contact points of the first permanent molars and the most vestibular point between the lower central incisors. Crowding is calculated as the difference between tooth size and arch length [13]. The amount of space required determines subsequent space management plans.

Additionally, lateral cephalograms can be used to assess skeletal parameters contributing to dental crowding, such as effective maxillary and mandibular length, mandibular plane angle, Y-axis, lower anterior face height, and dental parameters such as axial inclination of the lower incisor, inclination of the lower incisor to the mandibular plane, and interincisal angle [14]. Three-dimensional CT imaging provides better frontal and three-quarter profile data for diagnosis and allows precise measurements to be taken. Patano et al. emphasized that accurate diagnosis, through X-rays, cephalometric analysis, study models, and CBCT, is crucial for treatment planning [5]. While some studies argue that two-dimensional lateral cephalometric imaging and profile photos are insufficient for accurate diagnosis, CBCT is recommended as an adjunct to improve diagnostic accuracy [5].

The decision to manage space - whether by recovering or maintaining it - depends on the degree of crowding, the arch length to tooth material discrepancy, and the dental age of the patient. To prevent or intercept crowding, dentists should perform a comprehensive assessment of the malocclusion before formulating a management plan [5].

Space Maintainers

Primary dentition plays an essential role in ensuring the proper eruption of permanent teeth, along with supporting a child’s growth and development [15]. Premature loss of deciduous teeth can disrupt the alignment of permanent teeth, leading to space loss and malocclusion. In such cases, preserving leeway space is critical to maintaining proper arch integrity [5]. A proactive treatment approach with space maintainers becomes essential when primary teeth are lost prematurely, as these appliances help prevent complications such as crowding or the narrowing of the dental arch [15].

The Royal College of Surgeons highlights specific situations where space maintainers are particularly beneficial as follows: the loss of a primary molar in severely crowded arches where premolar extraction alone cannot alleviate crowding, or the loss of a second primary molar in cases of spaced arches [16]. Before deciding on space maintainer placement, a comprehensive assessment of the patient’s oral health is crucial. Factors such as caries risk, oral hygiene, and the child’s ability to cooperate must be evaluated. Space maintainers are not recommended for patients who struggle to maintain good oral hygiene, as plaque accumulation around the appliance may lead to further oral health complications. Stabilizing existing caries, ensuring adherence to a non-cariogenic diet, and conducting routine follow-ups are also critical to the success of space maintainers [17].

Parental negligence regarding space problems often leads to delayed clinic visits, leaving little room for conservative management. This can necessitate tooth extractions and space management strategies to prevent further complications like space closure or arch length narrowing [18,19]. Early intervention with space maintainers can prevent adjacent teeth from drifting into the vacant space and ensure the normal eruption of permanent teeth [19]. Ideally, space maintainers should be placed immediately following the premature loss of a primary tooth, as tooth migration can begin within six months of the loss [4].

Patient compliance plays a pivotal role in the effectiveness of space maintainers. A cross-sectional study conducted in Saudi Arabia surveyed 392 participants to assess parental knowledge of space maintainers and oral hygiene. The study revealed that only 43.3% of parents had received information about these devices, and 39.5% were unaware of their purpose. This underscores the critical role of dentists in educating parents and utilizing tools such as visual aids, radiographic images, and dental models to improve awareness [3]. Similarly, Ramadhani et al. highlighted the importance of parental education and identified pain and discomfort as primary reasons for low compliance rates among children using removable space maintainers [4].

Non-compliance, often due to discomfort or lack of understanding, is a significant barrier to the success of space maintainers. Addressing parental concerns and educating families about the importance of these appliances can improve outcomes and support better space management in pediatric patients [4].

Types of Space Maintainers

Various appliances can be used as space maintainers depending on the patient’s age, growth, dental arch development, and compliance with appliance wear. They are generally divided into removable and fixed space maintainers [2,4]. Fixed space maintainers are designed to preserve arch length. These are firmly inserted into the child’s mouth and are durable and well-tolerated by pediatric patients. These appliances require yearly visits to the dentist for inspection and cleaning [2]. Commonly used fixed space maintainers in pediatric patients include band and loop, crown and loop, lingual arch, and distal shoe maintainers [4].

Removable space maintainers, on the other hand, are typically indicated in cases of bilateral tooth loss or multiple extractions. They are easy to remove, allowing patients to maintain good oral hygiene and enabling routine inspection [20]. However, removable appliances have several disadvantages, including poor retention leading to frequent dislodgement, a higher likelihood of breakage, and less acceptance by pediatric patients. The most commonly used removable space maintainer is the Hawley retainer [4].

Patano et al. classified space maintainers into the following three categories: fixed unilateral appliances, fixed bilateral appliances, and removable partial dentures [5,9]. At present, conventional space maintainers are available in a wide variety of designs beyond the fixed or removable categories. As noted by Agarwal and Agrawal, they can also be classified as active or passive and functional or non-functional, depending on their mode of action and intended purpose [21]. Table 1 shows the types of space maintainers.

**Table 1:** Types of space maintainers.

Fixed unilateral space maintainer	Fixed bilateral space maintainer	Removable space maintainer
Band and loop	Nance palatal arch	Hawley appliance
Crown and loop	Transpalatal arch	Removable partial denture
Distal end shoe	Lower lingual holding arch	-
Fiber-reinforced composite resin	-	-

Band and loop space maintainers have been widely used as common permanent unilateral space maintainers. The band is cemented to the primary second molar, while the loop contacts the distal surface of the primary canine. While this appliance is simple and easy to use for both the dentist and the patient, conventional band and loop space maintainers have certain limitations, such as poor esthetics and a tendency to promote plaque retention [5]. Furthermore, it often requires multiple sittings for final delivery, although single-visit band and loop space maintainers are also available. The impression step, necessary after band formation, can be challenging for uncooperative child patients. Additionally, band displacement during cast pouring can lead to ill-fitting appliances, and its fabrication involves substantial laboratory work and time [22]. Recent literature has raised concerns about the longevity of this appliance, leading to recommendations for the use of crown and loop space maintainers instead. This variation of the band and loop appliance is specifically designed to maintain space following the loss of primary first molars [17].

The distal end shoe is another commonly used permanent unilateral space maintainer. It consists of a stainless steel wire that extends in front of the unerupted permanent first molar to guide it into position as it grows. However, this appliance can only be placed on one tooth [5]. Fiber-reinforced composite resin space maintainers are specifically used when the primary second molars are lost [17].

Bilateral space maintainers are employed when teeth on both sides of the mouth are lost. Examples include the transpalatal arch, Nance palatal arch, and lingual holding arch. The Nance palatal arch works by resisting the forward movement of posterior teeth, ensuring that space is preserved. This appliance has undergone various modifications, such as the Nance obturator, the modified Nance appliance for maxillary molar distalization, the modified Nance palatal arch for crossbite correction, and a version for anterior tooth replacement. In one patient, after the loss of the primary tooth, the Nance palatal arch was placed following the eruption of the first permanent molar. A long-term follow-up was conducted until the first premolar erupted to ensure successful treatment. This study concluded that fixed appliances remain an ideal solution for addressing space problems in pediatric patients, offering interim prostheses to restore both functional stability and esthetics when required [5].

A recent case study by Gupta et al. reported on a four-year-old child who experienced bilateral loss of primary maxillary molars due to severe decay [15]. Following careful evaluation, space maintainers were determined to be the safest option for preserving the extraction space. The prosthetic intervention was performed with a Nance appliance featuring functional components. The Nance palatal arch comprising bands adapted bilaterally to the posterior molars, a palatal wire arch, and an acrylic button positioned in the rugae palatine, proved effective. The free ends of the wire were soldered to the molar bands. The uniqueness of this appliance lies not only in its ability to maintain space but also in its functional advantages following premature tooth loss. Transpalatal arches are also frequently used in patients with bilateral primary molar loss [15]. The study concluded that this fixed appliance provided a reliable interim solution for the child’s functional and esthetic needs [15]. However, patient cooperation and positive behavior throughout treatment are critical for achieving success.

Habib et al. studied mandibular incisor crowding and highlighted the utility of the passive lower lingual holding arch (LLHA) [11]. This appliance maintains the position of the permanent first molars after the exfoliation of primary molars and canines, preventing their mesial drift into the extraction space. LLHA effectively alleviates mandibular incisor crowding during the transitional dentition stage. Their research included four children aged 11-13.5 years and focused on the appliance’s benefits for space maintenance. The Little Irregularity Index was used to assess the severity of mandibular incisor crowding before and after the LLHA application. The study concluded that the passive LLHA is an effective appliance for space maintenance during mixed dentition, as it significantly reduced mandibular incisor crowding over 20 months [11].

Removable space maintainers incorporate wire stops mesial and distal to the edentulous space and are often used as replacements for missing teeth, particularly anterior teeth. These appliances primarily aid in enhancing facial esthetics rather than maintaining space for lost teeth and are commonly referred to as removable partial dentures [5]. This category also includes the Hawley retainer. The success of removable appliances heavily depends on patient compliance and cooperation; extended periods of non-use can cause the appliance to no longer fit properly and lead to a loss of space [17]. Table 2 shows the indications, advantages, and disadvantages of different types of space maintainers.

**Table 2:** The indications, advantages, and disadvantages of different space maintainers.

Type of space maintainer	Indications	Advantages	Disadvantages
Band and Loop [5,17]	Premature loss of 1st or 2nd primary molar, with distal abutment tooth present for banding.	Used for both primary and permanent molar banding.	Cumbersome fabrication, time-consuming, costly; requires multiple visits; higher failure rate; more mesial drifting.
Crown and Loop [17]	Premature loss of 1st primary molar, with 2nd molar carious, requiring a crown instead of a band.	Offers better longevity compared to other space maintainers.	Same as above: cumbersome, costly, time-consuming, and higher mesial drifting.
Distal End Shoe [5,17]	Premature loss of 1st primary molar; tooth distal to this space remains unerupted.	Guides eruption of the first permanent molar when used early.	Complex and lengthy; requires patient cooperation.
Fiber-Reinforced Composite Resin [17]	Non-carious/restored surfaces for bonding on either side of the primary molar space.	Easy to fabricate; single-appointment appliance; reduced soft tissue damage; accessible to patients.	Requires rubber dam; higher chance of failure; technique-sensitive; long-term use is unproven.
Lingual Holding Arch [17]	Mandibular arch only; eruption of the first permanent molar is required.	N/A	Can hinder eruption of mandibular incisors; should be used after incisors erupt.
Nance Palatal Arch [5,15,17]	Maxillary arch only; includes a palatal acrylic button.	Maintains space in maxillary arch.	Potential for acrylic pad impingement in palatal soft tissue; difficult to clean.
Transpalatal Arch [15,17]	Maxillary arch only; used for multiple primary teeth loss or failure of unilateral maintainers.	Sustains transverse inter-molar distance.	Can cause tongue irritation; not suitable before the eruption of the first permanent molar.
Removable Appliances [5,17]	For multiple primary teeth loss.	Allows customization; removable and adjustable.	Requires two visits; poor patient compliance can lead to higher failure rates.

Survival of Space Maintainers

The utility of space maintainers has been widely accepted as an integral part of preventive orthodontics; however, an evidence-based approach is essential for selecting the most appropriate space maintainer for pediatric patients. The chosen appliance should be durable, functional, and capable of preserving arch length to ensure the normal eruption of permanent dentition [2]. The success of space maintainers depends on their overall survival rates, as well as their ability to effectively maintain space for the eruption of permanent teeth.

Fathian et al. conducted a study to evaluate the survival times and issues associated with laboratory-made space maintainers placed over seven years by a single pediatric dentist. The records of 235 patients were reviewed, revealing that 29% of the patients experienced good survival rates and great success with their appliances, 34% of the appliances remained in service, and 32% failed. Overall, 63% of the space maintainers either lasted their expected lifetime or were still in use between 1997 and 2003 [23].

Similarly, Moore and Kennedy studied the survival times of space maintainers used in patients with bilateral tooth loss over seven years [24]. A total of 482 space maintainers were evaluated, with 24% failures and 72% successes. The primary cause of failure was cement loss (60%), followed by solder breakage (10%), split bands (10%), and unspecified reasons (11%). The mean survival time was 20 months for lingual holding arches and 23 months for Nance appliances. This survey concluded that the majority of bilateral space maintainers (72%) demonstrated long-term success and high survival rates [24]. Furthermore, there is no space loss when a fixed unilateral space maintainer is retained intact, meaning the efficacy of these appliances largely depends on their longevity and survival rates. Multiple fixed unilateral space maintainers can be used for the same patient if there is tooth loss in different quadrants. However, their use should be limited to single-span edentulous areas due to the risk of breakage. The chances of failure are higher in patients who have lost two adjacent teeth [17].

Although extensive literature supports the long-term survival rates of various space maintainers, this success is attributed to continual modifications aimed at improving outcomes for both patients and dentists through advancements in materials, techniques, and technologies. Metallic space maintainers have been widely used for decades, but their long-term stability has often been questioned. Kirzioğlu et al. conducted a study involving 44 children who experienced premature loss of primary first molars [25]. Fiber-reinforced composite resin (FRCR) space maintainers were prepared using plaster models of the patients and directly fixed to the adjacent teeth. The study aimed to assess the clinical and radiographic survival rates of these space maintainers over 24 months or until failure. By the end of 12 months, 16.2% of the space maintainers were dislodged and classified as failed. At 24 months, a 52.2% success rate was recorded, while 31.8% of the space maintainers were removed due to the eruption of permanent teeth. The findings demonstrated that FRCR space maintainers exhibit commendable strength and durability, making them a viable alternative to metallic space maintainers [25].

The introduction of fiber reinforcement has significantly enhanced the utility of composite materials in restorative dentistry. Fiber-reinforced composites address several drawbacks associated with conventional space maintainers. These appliances require less fabrication time and are particularly beneficial for uncooperative or non-compliant pediatric patients due to their superior acceptance [18]. Garg et al. compared the clinical efficacy of conventional band and loop space maintainers with FRCR space maintainers [26]. The study included 30 children aged between five and eight years, and all participants had at least two deciduous molars in different quadrants that were either previously lost or indicated for extraction. An FRCR space maintainer was placed in one quadrant, while a conventional band and loop space maintainer was inserted in the other quadrant. Patients were followed up at one, three, and six months to evaluate the performance of both types of space maintainers. Using the chi-square test and Mann-Whitney U test, the study found that patients adapted better to FRCR space maintainers compared to band and loop appliances. Furthermore, the time required for FRCR space maintainers to achieve the intended function was significantly shorter. Failures in FRCR space maintainers were primarily due to debonding, as this is a technique-sensitive procedure, while failures in band and loop maintainers were mainly caused by cement loss. The success rate for fiber-reinforced composite resin (FRCR) space maintainers was recorded as 63.3% compared to 36.7% for band and loop space maintainers. This study concluded that FRCR maintainers could be considered a feasible alternative to conventional band and loop appliances, and in terms of clinical efficacy, they were found to be superior [26].

Conversely, a comparative evaluation by Tunc et al. examined the survival times and failure rates of the following three types of fixed space maintainers: band and loop, direct bonded, and fiber-reinforced composite resin [27]. They concluded that the mean survival time was highest for band and loop space maintainers (11.20 months) followed by directly bonded space maintainers (9.20 months). Fiber-reinforced composite resin space maintainers had the shortest survival time, at only 6.70 months [27]. Further studies have evaluated the clinical efficacy of band and loop space maintainers in greater detail and compared them with other conventional space maintainers. In one such study, Tyagi et al. examined 15 children aged between four and eight years, each having at least two fresh extraction sites of primary molars either contralaterally or bilaterally [27]. The study aimed to compare the efficacy of conventional band and loop space maintainers with conventional tube and loop, bonded tube and loop, and bonded band and loop space maintainers regarding gingival health, survival time, and patient and parent satisfaction. Conventional band and loop space maintainers were placed on one side, while either bonded loop, conventional tube and loop, or bonded tube and loop space maintainers were randomly inserted on the other side. After evaluations at one, three, six, and nine months, it was observed that conventional band-and-loop and tube-and-loop maintainers had a 100% survival rate, while bonded tube-and-loop maintainers had an 80% survival rate, and bonded band-and-loop maintainers showed only a 60% survival rate by the end of the study. Patients showed equivalent acceptance for all space maintainers, but on intergroup comparison, bonded space maintainers were more readily accepted. In terms of gingival health, conventional tube, and loop space maintainers demonstrated the best outcomes, whereas band and loop maintainers showed the worst [28].

It is imperative to compare different space maintainers before determining the best option to meet the patient’s needs. Bilateral fixed appliances, fiber-reinforced composite resin space maintainers, and band and loop appliances all show promising results in space management for pediatric dentistry. However, further studies with larger sample sizes and longer follow-up periods are required to investigate the long-term survival rates and efficacy of these space maintainers and support informed decision-making in clinical practice.

Space Regainers

The space regainer is an interceptive orthodontic appliance used to widen a narrow arch in cases of crowding or impaction by distalizing the posterior teeth to create space for the eruption of permanent teeth [4]. When space loss has already occurred and a space maintainer cannot resolve the issue, space regainers effectively regain lost space and prevent further malocclusion. In the maxillary arch, space is regained through the distalization of posterior teeth, while in the mandibular arch, devices such as lip bumpers or transpalatal arches are used. Although the transpalatal arch is primarily classified as a space maintainer, it can also function as a space regainer in specific cases by facilitating the distal movement and tipping of molars to create space and alleviate crowding [9].

The ideal age range for distalizing molars to regain lost space is between seven and 10 years [29]. Maxillary molars can be moved distally by 5-7 mm per side, while mandibular molars can only be distalized by 1-2 mm per side. Proper treatment planning is essential and involves using patient study models, periapical radiographs, clinical assessments, and cephalometric analysis [29]. Impaction of permanent teeth can lead to the early loss of primary teeth. While it is recommended to retain primary teeth in the arch until their natural exfoliation, if untimely loss occurs, it is critical to manage the negative consequences of the developing occlusion as early as possible [30]. In such cases, active tooth guidance with space-regaining measures may be necessary [19].

During the mixed dentition stage, interceptive orthodontics plays a crucial role by reducing the severity of the malocclusion and shortening the duration of complex orthodontic treatments. Invasive approaches, such as proximal stripping and serial extraction, are used to treat established malocclusions but these methods are irreversible. Therefore, non-invasive approaches, such as the use of fixed or removable space regainers, are often preferred for intercepting misalignments in the dentition [5,31].

Types of Space Regainers

There are various types of space regainers available, and the choice of appliance should be tailored to the specific needs of each patient.

Fixed space regainers: The pendulum appliance is indicated when a permanent molar migrates mesially into the space created by the early loss of primary molars. It is also used in the first phase of orthodontic treatment for unilateral or bilateral distalization of the first molar in the management of class II malocclusion [32].

The lip bumper is constructed using a heavy labial arch bar with an acrylic flange in the anterior region to prevent contact with the lower anterior teeth. This appliance is used in cases where bilateral movement is required, as it relieves lip pressure, which can then be redirected to distalize molars. This is achieved by incorporating loops in the archwire before it enters the buccal tube and by utilizing coil springs [32]. The lip bumper is specifically indicated for the lower arch to gain space or distalize molars, and its counterpart in the upper arch is the Denholtz appliance. In this appliance, molar bands are fitted on the permanent first molar, and molar tubes are welded onto the buccal side of each band. A labial archwire is engaged in the buccal tube, and an acrylic button is prepared in the labial vestibule. The lip bumper transmits forces from the lips directly onto the buccal aspect of the first molar and enables its distalization. It is also useful for uprighting mesially tipped molars to lengthen the arch [31].

The Herbst space regainer, also known as the open coil space regainer, is a reciprocal active appliance used in the mandibular arch after the eruption of the first premolar [32]. However, this appliance has certain limitations. For instance, it does not allow control over the axial inclination of the tooth being moved, which may result in tipping [33].

The anterior space regainer features a labial tube attached to the lateral incisor. A 0.014-inch round wire is inserted into an open coil spring and activated after three weeks [31]. As a small amount of space is gained, the wire is progressively changed to larger diameters, transitioning to a 0.016-inch round wire, followed three weeks later by a 0.018-inch wire, and finally to a 0.018 x 0.025-inch wire, leaving the coil spring in place for retention purposes. Subsequently, an acrylic pontic is fixed over the wire and coil spring using direct composite bonding [32,31].

The Gerber space regainer is advantageous due to its simple fabrication process, which can be completed directly in the mouth, minimizing laboratory work. This appliance utilizes a U-shaped assembly into which a U-shaped wire can be fitted. The wire is soldered onto the mesial aspect of the band. A compressed coil spring is fitted onto the U-shaped wire, which is then inserted into the U assembly. Once cemented onto the teeth, the ligature is cut and removed to activate the regainer [31,32].

The sectional arch technique is utilized in cases where the second molar has erupted. This appliance effectively regains lost arch length and allows for space recovery of up to 4 mm [32]. The Hotz lingual arch is indicated when permanent teeth move mesially rather than distal movement of mesial teeth, as well as in cases where there is sufficient space for the eruption of the permanent second molar. The permanent first molar is distalized using a wire spring attached to the lingual arch. Additionally, a loop is incorporated into the arch and gradually opened once a month to regain space. Adequate anchorage is needed to exert the necessary force for distalizing the first molar [31,32].

Removable Space Regainers

The jackscrew space regainer is used to regain lost space caused by drifting teeth into an edentulous area. It is activated regularly to exert consistent force against the banded teeth. A bilateral version of this appliance includes a coil-loaded lingual arch that passes through tubes soldered lingually onto molar bands, producing rapid results [32].

The slingshot space regainer derives its name from the distalizing force generated by elastic bands stretched along the lingual and buccal surfaces of the molar to be moved. To move the first permanent molar distally, hooks are attached to the buccal and lingual surfaces of the molar, and an elastic band is stretched between them. The force exerted by the elastic band facilitates distal movement, which is typically limited to 1-2 mm. The elastic band is replaced daily to maintain effectiveness [32]. The removable nature of this appliance necessitates periodic check-ups to assess patient compliance, detect breakages or distortions, and address any irritation to the soft tissues. If teeth begin to erupt beneath the appliance, portions of the acrylic base are removed to create space for proper eruption. Regular radiographic examination of developing permanent teeth is essential. The appliance is discarded once the successor teeth have erupted into their correct positions in the oral cavity [31].

The free-end loop space regainer consists of a labial arch wire for stability and retention, a back-action loop spring made of 0.0025 wire, and an acrylic base. The free end of the loop is periodically activated to achieve the desired movement of the tooth. This appliance exerts a light force on the targeted tooth and requires periodic monitoring to evaluate the magnitude of the force. In the lower arch, the shorter wire loop minimizes distortion during appliance insertion, making it effective and easy to use [31].

The split saddle or split block space regainer is commonly used in the lower arch to achieve distal movement of the first permanent molar. It is activated by flattening the bent portion of the wire that connects the split saddles of the acrylic base plate. The activator mechanism of this appliance is similar to fixed bridge therapy. However, the unilateral variant is not used in children due to the risk of accidental swallowing. The distal movement achieved with this appliance is typically limited to 1-2 mm [31,32].

Fixed space regainers are generally preferred over removable ones because they are more beneficial for patients and require less treatment time. They also ensure better patient compliance and allow for more efficient oral hygiene maintenance.

Modifications in Space Regainers

Although various indications for different space regainers have been highlighted in this review, some studies have explored using space maintainers as modifications to fulfill the same purpose as space regainers, thereby enhancing the efficiency of these appliances. Romano et al. presented two cases involving the ectopic eruption of permanent maxillary first molars treated with a modified Nance palatal arch to facilitate distal movement [34]. In these cases, the appliance was designed such that the wire was soldered to the band and positioned distally to support an elastic chain attached to a bonded button on the occlusal surface. After a few months, the ectopically erupted permanent maxillary first molars were successfully corrected, preserving the adjacent primary teeth and restoring function. The mechanism of the Nance palatal arch involved distalization, which effectively regained lost space in the maxillary arch [34].

A comparative study evaluated the effectiveness of the lip bumper and transpalatal arch as space regainers. It concluded that the lip bumper resulted in more significant space regain compared to the transpalatal arch. The lip bumper achieves superior efficiency by utilizing the muscles of the lower lip to apply continuous distalizing force on the mandibular first molars, while the tongue exerts opposing labial force on the mandibular incisors. Additionally, prefabricated lip bumpers with thicker shields demonstrated greater efficiency in increasing arch length compared to those fabricated from stainless steel round wire. This enhanced performance was attributed to the greater contact area with the lower lip muscles, which exerted stronger distalizing forces [9].

In complex cases, alternatives to conventional space regainers may be needed. In some situations, functional appliances may serve as space regainers. For example, a 13-year-old patient with a class II, division 1 malocclusion on a class II skeletal base required orthodontic space creation. Options such as molar distalization, proclination of incisors, interdental stripping, extraction, or arch expansion were considered. Expansion was the only viable option, as it is estimated that 0.5-0.7 mm of space can be gained for every 1 mm of posterior arch expansion. A twin block appliance was used in this patient, and the space gained after the expansion was utilized to adjust for the missing central incisor. This study demonstrated that functional appliances like the twin block can effectively create space by expanding the maxilla while simultaneously correcting the sagittal relationship [35].

Modifications in space regainers often involve incorporating space maintainers or functional appliances and adapting their designs to improve functionality, patient comfort, and ease of use. These modifications aim to address the limitations of conventional space regainers and strike a balance between appliance effectiveness and patient compliance.

Contemporary Trends and Future Prospects

This review discusses conventional space maintainers in detail, highlighting their effectiveness in various use cases. In recent years, contemporary trends such as three-dimensional (3D) printing, also known as desktop fabrication, have emerged in pediatric dentistry. This technology is based on computer-aided design and computer-aided manufacturing (CAD/CAM) and utilizes standardized materials to fabricate customized 3D objects through a layer-by-layer approach [36]. Several studies have been conducted to explore this evolving technology and its application in pediatric dentistry.

One case study demonstrated the revolutionary use of 3D-printed bands and loop space maintainers. By incorporating CAD/CAM technology, clinicians were able to design patient-specific prostheses to guide the eruption of teeth [37]. This case involved an autistic child, and the 3D-printed space maintainers proved advantageous due to their high precision, diagnostic accuracy, efficient treatment planning, and rapid production. The reduced chair-side time also minimized aerosol exposure and made the procedure more suitable for children with special needs [37]. The process began with pouring the primary cast dentition to trial the design of the 3D-printed space maintainer through digital scanning and design. The retrieved cast was scanned using a 3D digital dental scanner, and the band and loop were designed with 3Shape dental software (Copenhagen, Denmark: 3Shape). Fabrication was carried out using direct metal laser sintering with a cobalt-chromium (Co-Cr) base alloy, chosen for its cost-effectiveness compared to titanium alloys, and the Shinning 3D DMLS printer (Hangzhou, China: Shining 3D) was used. After finishing and polishing, the 3D-printed space maintainer was tested in the patient’s mouth and cemented with glass ionomer cement. Post-procedural instructions included avoiding hard foods for 30 minutes [37].

In addition to metals, other biomaterials are available for 3D dental material fabrication, such as ceramics, hydrogels, thermoplastics, and polymer-based materials [38]. For 3D-printed dental prostheses, titanium and Co-Cr alloys are preferred due to their superior corrosion resistance, hardness, and bonding capacity [39,40].

Another study by Guo et al. combined digitization with pediatric dentistry by using scanning techniques and laser medical image reconstruction to construct a digital model of dentition defects [41]. This research focused on producing 3D-designed removable space maintainers. Using 3Shape software, they compared the following two fabrication methods: polyetheretherketone (PEEK) and conventional techniques. Conventional removable space maintainers, though widely used, have drawbacks such as complicated manufacturing, technique sensitivity, and significant individual variation. Additionally, conventional methods often involve self-curing resin, which can shrink during polymerization and cause poor appliance fit. In contrast, PEEK removable space maintainers were found to be more accurate, offering superior fit and precision. As a special engineering plastic, PEEK is highly rigid, tough, and biocompatible, with resistance to temperature, corrosion, and radiation. It is used in fixed prosthodontics for crowns, bridges, healing abutments, and removable partial denture scaffolds [40]. The study concluded that digitally designed removable space maintainers are superior to conventional ones. Three-dimentional variation analysis revealed that CAD/CAM-manufactured space maintainers were highly suitable for clinical application, as the mean distance and standard deviation of the PEEK group were significantly smaller than those of the conventional group (p < 0.01). The PEEK design was more stable and durable [41]. Ierardo et al. similarly concluded in a pilot study that PEEK is a highly suitable material for constructing removable space maintainers using dental CAD/CAM systems [42].

Other applications of 3D printing in pediatric dentistry include presurgical nasoalveolar molding, crossbite correction, myofunctional appliances, orthodontic brackets, regeneration of teeth and supporting structures, and zirconia crowns. However, the use of this technology in pediatric dentistry requires further investigation [37].

As described above, there is a wide variety of space maintainers and space regainers available, providing effective space management solutions for pediatric patients. Though these appliances are highly useful, they have certain limitations. To address these, an innovative design called the "tube and loop" space maintainer (Nikhil appliance) has been researched. This appliance offers several advantages, including the elimination of certain fabrication steps such as impression making, band transfer, and laboratory procedures like soldering. It is time-saving and serves as a good alternative to the band and loop space maintainer. In one case study, a seven-year-old female patient visited the clinic with a chief complaint of pain and swelling in the maxillary left posterior region for the past five days. Clinical examination revealed that the primary maxillary left second molar was grossly decayed. Due to the poor prognosis of the tooth, it was extracted, and a "tube and loop" appliance was planned and delivered to the patient. Eight months later, the appliance demonstrated its success - the permanent successor (maxillary left second premolar) had erupted uneventfully in its original position without any space discrepancy. This novel appliance has been recommended as an efficient solution for challenging space management cases, as it offers advantages over conventional space maintainers [22]. Similarly, other innovative approaches to traditional patient presentations have significantly improved space management strategies.

Among pediatric patients, dental fear and anxiety remain significant challenges for dentists. Longer appointments often heighten children’s apprehension, and the success of treatment heavily relies on managing their behavior. Placing preformed bands reduces chair-side time and helps manage the child’s behavior [43]. A case report demonstrated the use of performed space maintainers as an alternative to fixed space maintainers to minimize chair-side time and the number of appointments in an apprehensive patient [20]. Goswami et al. reviewed prefabricated band and loop space maintainers as a novel innovation to overcome the drawbacks of conventional band and loop maintainers [43]. In their case series, five pediatric patients were treated using prefabricated band-and-loop space maintainers, which required only one appointment, involved minimal laborious work, and were affordable [36]. Seita et al. reported a higher success rate of 92.3% with prefabricated space maintainers compared to 86.7% for conventional types at three, six, and nine months [44]. Similarly, Tahririan et al. found that both conventional and prefabricated band and loop maintainers demonstrated equal success rates of 100% at one and three months, which decreased to 92% by nine months [45].

The use of artificial intelligence (AI) in pediatric dentistry has also been explored. Aksoy et al. investigated the use of AI chatbots to provide space maintainer-related information to pediatric patients and their parents [46]. Their methodology involved forming 12 space maintainer-related questions based on current guidelines and directing them to ChatGPT-3.5 (San Francisco, CA: OpenAI) and ChatGPT-4. The responses were assessed for quality, reliability, readability, and similarity to existing literature using tools such as EQIP, DISCERN, FRES, FKRGL calculation, GQS, and the Similarity Index. Results showed outstanding quality from ChatGPT-3.5 and good quality from ChatGPT-4, with mean scores of 4.58 ± 0.515 and 4.33 ± 0.492, respectively. The study concluded that AI-based chatbots could be a valuable tool for providing dental information and assisting in pediatric space management and treatment planning [46].

Overall, digitization in pediatric dentistry has allowed clinicians to shorten treatment durations while enhancing diagnostic accuracy and treatment planning approaches. The emergence of 3D printing represents a step toward the future of "smart" and "green" pediatric dentistry [37]. However, long-term studies are needed to evaluate the performance and success rates of both conventional and innovative space maintainers. Further research is also necessary to explore new design ideas, advanced materials for manufacturing, and the integration of AI in space management strategies for young children.

## Conclusions

The best space maintainer is a well-established primary dentition. Dentists should therefore always make an effort to preserve the natural dentition, which requires educating the pediatric patient and their parents to create awareness regarding space problems in the mixed dentition stage and their negative impact on permanent dentition. If these natural space maintainers are prematurely lost, it is important to promptly introduce a space management strategy for a child’s functional and esthetic well-being. Space management can be done through space maintainers and space regainers. Space maintainers are part of a preventive treatment regimen and help maintain the space created by the untimely loss of primary teeth. Space regainers play an important role in interceptive orthodontics and recreate the lost space by distalisation of posterior teeth. As both types of appliances have conventional as well as contemporary variants, it is the clinician who decides what works best for the individual patient. This review has provided an overview of the role of space maintainers and space regainers in the management of space problems. The survival rates and efficacy levels of the appliances are clear indicators of success. Moreover, contemporary trends and modifications in these appliances have enhanced their usability by increasing their range of action. Further case studies are needed to individually evaluate the utility of each appliance in various patients, and more long-term studies are required to investigate novel approaches in the management of space problems in pediatric patients.
